# Robust Flow Estimation Algorithm of Multichannel Ultrasonic Flowmeter Based on Random Sampling Least Squares

**DOI:** 10.3390/s22197660

**Published:** 2022-10-09

**Authors:** Zhijia Xu, Minghai Li, Yuqiang Han, Xin Li, Guangmei Shi

**Affiliations:** 1 Institute of Systems Engineering, China Academy of Engineering Physics, Mianyang 621999, China; 2 College of Geology Engineering and Geomantic, Chang’an University, Xi’an 710054, China

**Keywords:** multi-path ultrasonic flowmeter, random sampling consensus algorithm, least squares

## Abstract

The multi-path ultrasonic flowmeter is widely used in engineering practice, and the flow algorithm is important for its accuracy. The least-squares estimation method is simple and efficient and has good engineering application value. In practical applications, noises are inevitably introduced to the measurement process because of the flowmeter itself or flow-field interference. The results of classical least squares will deviate from reality because it lacks robustness. In this regard, two flow algorithms of multi-path ultrasonic flowmeter are proposed based on least-squares and random-sampling consensus algorithms, which are widely used in the image field. The two algorithms can resist gross errors effectively by avoiding the interference of external points in the sampling points. To verify the effectiveness of the proposed algorithms, we take the double-bend flow field, which is a typical damaged flow field in engineering, as the research object, and then we compare the four algorithms. It can be seen that the two flow algorithms have higher accuracy and better robustness in the presence of interference noise.

## 1. Introduction

With the development of social economy, the accurate measurement and billing of energy-carrying fluids, such as hot water, gas, and oil, are of great significance to people’s daily life. In nuclear power plants, the accurate measurement of the flow of main feedwater will greatly improve the efficiency of nuclear power plants and is important for the long-distance transmission of gas and gasoline.

Flowmeters can be classified into mechanical flowmeters, electromagnetic flowmeters, vortex flowmeters, and ultrasonic flowmeters. The ultrasonic flowmeter is widely used in various fields because of its convenient installation, wide range ratio, low flow startup, and high accuracy, even in pulsating flow [1]. The time-difference ultrasonic flowmeter is the most widely used. It indirectly calculates the linear velocity of the propagation channel through the time difference between the forward and reverse flow propagation based on the influence of the flow velocity on the sound velocity; it then calculates the surface velocity through the linear velocity or velocities to finally obtain the flow. Based on the number of channels, this type can be divided into the single-path ultrasonic flowmeter and multi-path ultrasonic flowmeter. In theory, a more accurate flow can be obtained when more velocity profile information is known; thus, the multi-path ultrasonic flowmeter has higher accuracy under the same conditions. How to arrange the sound channel, how to determine the weight coefficient, and how to calculate the flow through the measured linear velocity are the flow-algorithm problems that should be resolved.

The two methods of flow algorithms are integration and optimization. Many integration methods based on Gauss integral, such as Equal Tab, Gauss Jacobi [2,3], OWICS [4], and Tailore [5], are widely used in multi-path ultrasonic flowmeters. Tresch [6] comprehensively analyzed the first three methods and reached a conclusion that their acoustic channels are fixed and more suitable for ideal flow fields. By using Gauss integration, Zheng [7] proposed an improved numerical integration method for non-ideal flow fields. To a certain extent, more appropriate channels and weights can be selected according to specific flow-field conditions. Qin [8] proposed a method based on the generalized inverse matrix (GIM) to make up for the shortcomings of Gaussian integration (only parallel channels can be selected and the weight coefficients are fixed). This method can be applied to any channel arrangement and can improve the accuracy and robustness of the flowmeter. In general, integration aims to obtain channel arrangement and weight coefficients by applying reasonable numerical integration on the fixed velocity profile model. Most of the integration methods study the channel arrangement and weight coefficients by assuming a fixed velocity profile model. For this reason, constructed and reconstructed velocity profiles are important. For example, Moore [9] proposed several classical velocity profiles based on the classical power-law velocity profile and considering factors such as asymmetry and lateral velocity; the effectiveness of the velocity profiles has been verified by many scholars. Rychagov [10] also proposed to reconstruct a flow field based on Abel’s transformation.

On the premise of the fixed-flow algorithm model, various optimization algorithms can be used to determine optimal weight coefficients and improve the accuracy. Many methods can be used to determine the optimal weight coefficients of the flow algorithm model by using least squares, least squares with forgetting factor, nonlinear least squares, and Levenberg–Marquardt algorithms [11,12,13]. In addition, research on constructing a deep learning model for the weight coefficients of multichannel ultrasonic flowmeters has progressed with the development of intelligent algorithms, such as the ANN method [14], support-vector-machine method [15], PSO–SVM method [16], and adding a recognizer before importing a certain deep-learning model [17,18]. The deep-learning algorithm has great prospects due to its high precision. However, at present, it is inconvenient to use in practical applications because weight parameters calculated by deep learning vary from dozens to thousands or even more according to the depth model; moreover, the parameters are sensitive to the velocity profile, for which it is difficult for the project to meet its requirements. Even if a recognizer is added, including all types is difficult, and the recognition sensitivity and reaction time need to be further studied.

The most commonly used multichannel flow algorithm model is still $\;v_{M}=\overset{n}{\underset{i=1}{\sum }}w_{i}v_{Mi}$. In this classical linear model, the least-squares or weighted-least-squares estimation method is simple and effective. However, in consideration of the reasons associated with the flowmeter itself or the flow-field interference, the measured value of the multi-path flowmeter may have noise or gross error. The classical LS (least squares) algorithm cannot resist the error, while the WLS (weighted least squares) can determine the weights through the LS residual but cannot distinguish small observation anomalies. This paper proposes the RLS (least-squares method based on random sample consensus algorithm) method with random sampling and least squares based on the LS algorithm and the idea of random sampling consistency that is widely used in image denoising to improve the accuracy and robustness of least squares in flowmeter measurements. However, in practical application, determining the threshold value for judging the outer point that requires repeated debugging is difficult. As such, RMLS is also proposed to obtain the optimal LS observation sample through adaptive iteration, using the median deviation of random samples. The effectiveness of RLS and RMLS was verified by using a double-bend pipe as the research object. Data acquisition was carried out by CFD software based on the four-path ultrasonic flowmeter, and random Gaussian noises were added. Monte Carlo simulation experiments were carried out to compare the flow-estimation accuracy and robustness of the four estimation algorithms. The results indicate that RLS and RLMS have higher accuracy and better robustness in the presence of noise.

## 2. Measurement Model and Data Acquisition

**Figure 1:**
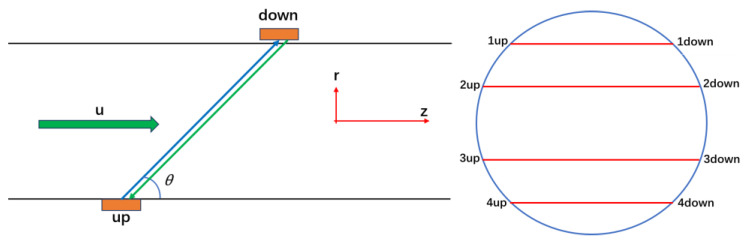
Schematic diagram of time-difference measurement.

In this work, a double-bend pipe was taken as the research object, and the four-path ultrasonic flowmeter based on time-difference method was studied. The measurement schematic diagram is shown in Figure 1. The time-difference ultrasonic flowmeter calculates the linear velocity of the flow at acoustic path through the difference in the transit time between upstream and downstream. The four-path ultrasonic flowmeter calculates the surface average velocity through the four measured linear velocities to measure the flow.

In this study, the optimization algorithm was used to convert line-average velocity to the surface-average velocity. The data of line and surface velocities of the velocity profile were collected for optimization training to determine the model parameters. The quantity and quality of the collected data directly affect the accuracy of the optimization model. To obtain good data in a more flexible manner, we used computational fluid dynamics (CFD) method. The accuracy of data with the CFD method has been verified in many literatures [19,20]. Noise data are synthesized by adding Gaussian noise to the original data collected by the CFD method.

**Figure 2:**
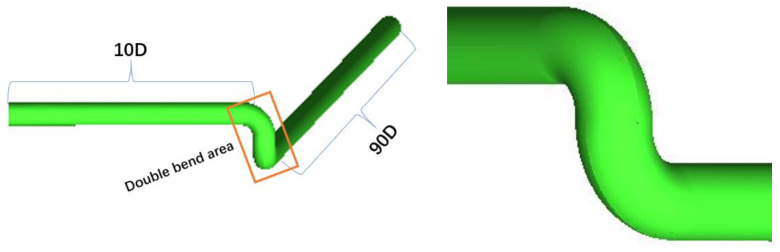
Global and local views of geometric model.

Taking the double bend as the research object and considering the flow measurement of the velocity profile near the bend and relatively far from the bend, we set the ultrasonic flowmeter measurement surface at x = 15, 50, and 300 cm. The origin of the three-dimensional coordinate system is located at the center of the elbow-region inlet. Given that we only studied the straight pipe after the double-bend area, we can take the section at the outlet of the elbow as the reference plane. The reference plane outlet is located at x = 10 cm, which means that the fluid enters the straight pipe from x = 10 cm, and the distances between x = 15, 50, and 300 cm and the double bend are 0.5D, 4D, and 29D, respectively. Geometric modeling and mesh division are carried out by ICEM [19]. The pipe diameter is D = 10 cm, and the length of 90D is set after the double bend, where x = 910 cm. The specific geometric model is shown in Figure 2. The distance from the inlet to the double-bend area is 10D, and the distance from the outlet to the double bend area is 90D.

**Figure 3:**
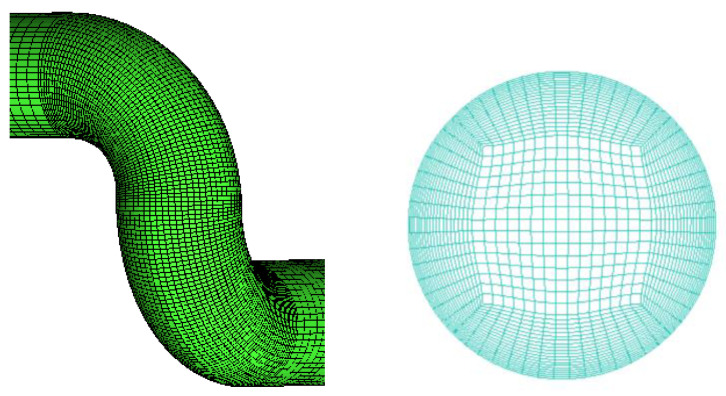
Gridding diagram.

A big velocity gradient exists in the boundary layer near the wall because of the wall and viscosity. The boundary-layer meshes are set as local refinement to accurately capture the flow of the boundary layer. The mesh is densified in this region because the curvature of the double-bend region is large, and the geometric complexity has a great impact on the flow field. The whole pipe is divided into structured meshes and then transformed into unstructured meshes imported into fluent for calculation to improve the mesh quality. The mesh quality is above 0.65. Figure 3 shows the grid-division diagram of the elbow part and the cross-section of the pipe.

**Figure 4:**
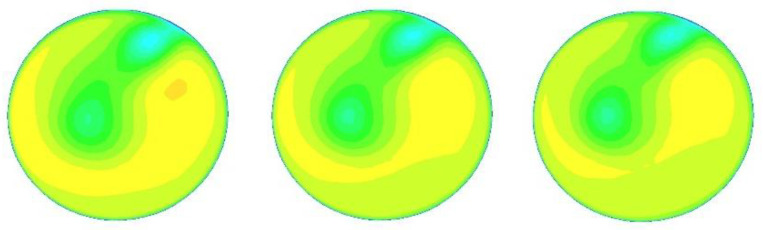
Velocity cloud diagrams at 2, 4, and 6 m/s at x = 15 cm (from left to right).

**Figure 5:**
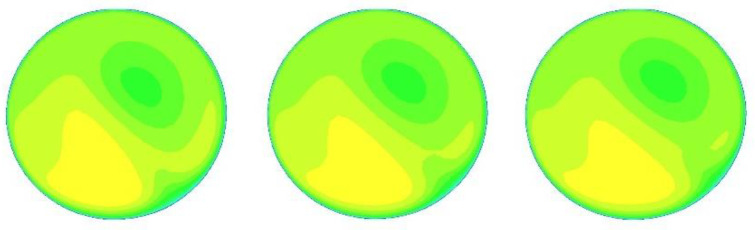
Velocity cloud diagrams at 2, 4, and 6 m/s at x = 50 cm (from left to right).

**Figure 6:**
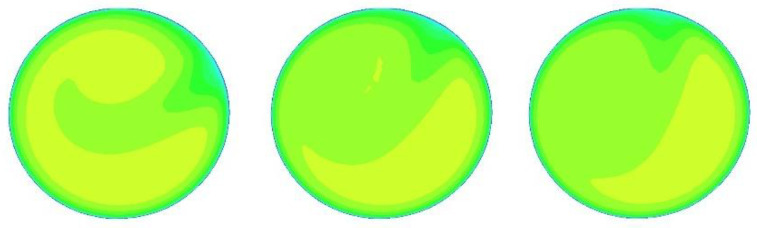
Velocity cloud diagrams at 2, 4, and 6 m/s at x = 300 cm (from left to right).

The CFD software was run 25 times with 25 different inlet velocities (1, 1.2, 1.5, 1.7, 2, 2.2, 2.5, 2.7, 3, 3.2, 3.5, 3.7, 4, 4.2, 4.5, 4.7, 5, 5.2, 5.5, 5.7, 6, 6.2, 6.5, 6.7, and 7 m/s) to provide sufficient data for optimization. For other boundary conditions, the outlet is set to “outflow”, and the pipe wall is set to “wall”. The k-e realizable model [21] is selected due to the complex flow field of the double bend to ensure the accuracy of the flow-field calculation. A grid independence experiment is conducted, and the results are consistent when the number of grids reaches 1.2 million. In this work, the number of grids is about 1.67 million, which is sufficient to ensure the authenticity and validity of the results. The velocity cloud images of x = 15, 50, and 300 cm at speeds of 2, 4, and 6 m/s are shown in Figure 4, Figure 5 and Figure 6, below. 

Serious deformation occurs in the flow field after the bend, and the flow field is obviously rotating from x = 15 cm to x = 50 cm to x = 300 cm. However, the profile velocity clouds have certain similarities under different velocities at the same position, as shown in Figure 4, Figure 5 and Figure 6. The research of $w_{i}$ of the multichannel ultrasonic flowmeter at a fixed position is meaningful because of this similarity, indicating that corresponding weight coefficients can be selected according to the position from the double bend.

## 3. Methods

### 3.1. Linear Model of Flow Algorithm

The most widely used linear model of the flow algorithm for a multichannel ultrasonic flowmeter can be described as Equation (1):

(1) $$v_{M}=\overset{n}{\underset{i=1}{\sum }}w_{i}v_{i}$$

where $v_{M}$ denotes the average velocity of the profile, and n is the number of channels. In this study, n=4, $v_{i}$, and $w_{i}$ are the measured velocity and its corresponding weight coefficients at any channel. The specific parameterization of Equation (1) is as follows:

(2) $$V_{M}=\left[\begin{array}{c} v_{M1}\\ v_{M2}\\ \vdots \\ v_{Mn} \end{array}\right],\;\;W=\left[\begin{array}{c} w_{1}\\ w_{2}\\ \vdots \\ w_{n} \end{array}\right],\;\;V=\left[\begin{array}{cccc} v_{11} & v_{12} & \cdots & v_{1n}\\ v_{21} & v_{22} & \cdots & v_{2n}\\ \vdots & \vdots & \ddots & \vdots \\ v_{m1} & v_{m2} & \cdots & v_{mn} \end{array}\right]$$

where $v_{ij}$ denotes the measured velocity of the i-th profile and the j-th channel. The flow model can be written as Equation (3):

(3) $$VW=V_{M}$$

The above formula is a linear model. The optimization problem is related to obtaining the accurate value of W. Many existing algorithms are used to solve problems; these algorithms, such as the least squares (LS) and Gauss–Newton-based nonlinear optimization method, can obtain satisfactory results if the measured velocity (V) is sufficiently accurate. However, noise or measured gross error may affect the reliability of the traditional flow-estimation algorithm due to the interference of the flow field or the inaccuracy of the ultrasonic flowmeter. Thus, we propose a robust flow algorithm of multichannel ultrasonic flowmeter based on random sampling least squares. The traditional LS-based flow-estimation algorithm is introduced firstly, and the proposed robust flow-estimation algorithm is presented in this section. 

### 3.2. Traditional LS-Based Flow-Estimation Algorithm

The LS method is a standard approach in regression analysis to approximate the solution of overdetermined systems by minimizing the sum of the squares of the residuals made in the results of each individual equation, so the sum of squares of errors between the data obtained by the function and the true value is minimized. 

For uniform description, the flow calculation model $v_{M}=\overset{n}{\underset{i=1}{\sum }}w_{i}v_{i}$ is presented as Equation (1). The LS-based flow-estimation algorithm can be adopted to convert the problem into Equations (4) and (5):

(4) $$\begin{array}{c} J\left(W\right)=\begin{array}{c} \left||VW-V_{M}|\right| \end{array} \end{array}$$

(5) $$\hat{W}=argmin\left(J\left(W\right)\right)$$

where $\begin{array}{c} \left||\cdot |\right| \end{array}$ represents the 2-norm, and the extremum of *J(W)* differential can be obtained as follows:

(6) $$V^{T}VW=V^{T}V_{M}$$

According to the properties of the matrix, when $V^{T}V$ is a nonsingular matrix, W has a unique solution, which is as follows:

(7) $$\hat{W}={\left(V^{T}V\right)}^{-1}V^{T}V_{M}$$

For LS, each group of data is of equal accuracy by default, but noise is difficult to avoid due to flow-field interference, instrumentation, and other problems. The traditional least-squares method will have difficulty maintaining its accuracy level.

Thus, weighted least squares (WLS) is usually used to solve the above LS problem, which adds a weight matrix (M) to the LS, so the model becomes closer to reality. The key is how to obtain the weight matrix of WLS. In general, the LS-estimated Equation (7) is taken as the iterative initial value, ${\hat{W}}^{\left(0\right)}$, to obtain the initial residual vector, e.

(8) $$e=V\hat{W}-V_{M}$$

Then e is normalized to obtain u, and Equation (9) is used to obtain the initial weight:

(9) $$M=\frac{\varphi \left(u_{i}\right)}{u_{i}}$$

where $M_{i}$ is the diagonal element of M. In this study, φ can be obtained as follows:

(10) $$\varphi \left(u_{i}\right)=\frac{tanh\left(u_{i}\right)}{u_{i}}$$

where tanh is the hyperbolic tangent function. Then we calculate the following:

(11) $${\hat{W}}^{\left(1\right)}={\left(V^{T}MV\right)}^{-1}V^{T}MV_{M}$$

${\hat{W}}^{\left(1\right)}$ replaces ${\hat{W}}^{\left(0\right)}$ to find a new residual, and so on. Iteration is continued until the difference between $\hat{W}$ in two adjacent steps is less than the predetermined value:

(12) $$max\left|{\hat{W}}^{\left(\tau \right)}-\begin{array}{c} {\hat{W}}^{\left(\tau -1\right)} \end{array}\right|<\varepsilon$$

In this paper, the threshold, *ε*, is set to $10^{-5}$.

We give the specific sketch of the WLS-based flow-estimation algorithm as Algorithm 1.
**Algorithm 1: WLS-based flow estimation****Input:** Measured velocity matrix, V, and profiled average velocity vector, $V_{M}$. **Calculation process:** 1. According to Equation (7), calculate the initial value of $\hat{W}$; 2. According to Equation (8), calculate the residual vector, e; 3. According to Equation (9), calculate the weight matrix, M; 4. According to Equation (11), recalculate the matrix, $\hat{W}$; 5. If Equation (12) is satisfied, exit the program; otherwise, repeat Steps (2) to (4); 6. Use Equation (11) to obtain the optimal resolution of $\hat{W}$.

### 3.3. Proposed Robust Flow Estimation Algorithm

WLS uses a posteriori residual to determine the weight of the observation value, which can enhance the noise resistance of the flow estimation to a certain extent. However, the algorithm is sensitive to the residual value of different observation anomalies and can easily fall into local optimization. To obtain better noise immunity and improve the engineering adaptability of the ultrasonic flowmeter, we propose the use of least-squares method based on the random-sample-consensus algorithm (RANSAC) for flow calculation. This method is called RLS. 

A data subset is randomly extracted from the whole dataset, in which only four velocity profiles exist for the four channels. The LS method is conducted for the extracted subset to obtain a set of model parameters ($\hat{W}$). With every calculated $\hat{W}$, we can gain the distances of the whole data by using Equation (13):

(13) $$D=\frac{\left|\begin{array}{c} w_{1}v_{1}+w_{2}v_{2} \end{array}+w_{3}v_{3}+w_{4}v_{4}\right|}{\sqrt{w_{1}^{2}+w_{2}^{2}+w_{3}^{2}+w_{4}^{2}}}$$

According to the calculated distances, inner and outer points can be easily distinguished with a predetermined threshold ($\varepsilon_{d}$). If the distances are less than $\varepsilon_{d}$, then the data point belongs to inner points (inliers); otherwise, it belongs to outer points (outliers). 

The outer points generally have large noises. If only the inner points are used for LS estimation, then a robust resolution can be obtained. The key of RLS is how to select an optimal data subset, which is equivalent to the maximum number of its corresponding inner points; in general, it can be determined by giving a distance threshold ($\varepsilon_{d}$) and maximum sampling iteration times (k) in the program. The formula for distinguishing inliers and outliers is as follows:

(14) $$N_{i}=\{\begin{array}{cc} 1 & D\leq \varepsilon_{d\;}\;\\ 0 & D>\varepsilon_{d\;} \end{array}$$

where $N_{i}=1$ means the inliers, conversely outliers. For the total data (points), the number of inliers can be easily counted. If the optimal subset is selected, then the number of inliers is expected to be the maximum. 

The performance of RLS for flow estimation strongly relies on the empirical parameters of $\varepsilon_{d}$ and k. To improve the adaptive ability of the RLS algorithm, we can use the following theoretical formula to compute the maximum sampling iteration time, k:

(15) $$1-c={\left(1-q^{n}\right)}^{k}$$

where *c* is the confidence and set to 0.98 in this study, *q* is the proportion of outliers in the dataset, *n* is the number of sampling points, and *k* is the maximum sampling iteration times. From Equation (14), we can obtain the following:

(16) $$k=\frac{\log\left(1-c\right)}{\log\left(1-q^{n}\right)}$$

The specific sketch of the RLS-based flow-estimation algorithm is as Algorithm 2:
**Algorithm 2: RLS-based flow estimation****Input:** Measured velocity matrix, V, and profiled average velocity vector, $V_{M}$. **Calculation process:** 1. For the t-th iteration, randomly generate the subset data, namely $V\left(s_{t}\right)$ and $V_{M}\left(s_{t}\right)$; 2. According to Equation (7), calculate the $\hat{W}\left(s_{t}\right)$; 3. According to Equation (13), calculate the distance, D, of the total data; 4. According to Equation (14), statistically determine the number of inliers, namely $n\left(s_{t}\right)$. 5. According to Equation (16), calculate the maximum sampling iteration time, k; 6. If iteration time t<k, count the maximum $n\left(s_{i}\right)$ and corresponding subset data; 7. Repeat Steps (1) to (6) at t=k to obtain the optimal subset data, $V\left(s_{i}\right)$ and $V_{M}\left(s_{i}\right)$. 8. Based on the selected optimal subset and according to Equation (7), calculate the $\hat{W}$. **Output:** Velocity weight coefficient vector resolution, $\hat{W}$.

In theory, the RLS method introduces the random-sampling-consistency method (RANSAC) into least squares (LS). The parameters are optimized continuously with the whole dataset divided into inner points and outer points through a reasonable threshold, and noise pollution can be effectively eliminated. The calculation results should be very robust, and measurement results with certain accuracy can be obtained even in the case of partial path-measurement failure.

The distance threshold ($\varepsilon_{d\;}$) of RLS needs to be adjusted and determined repeatedly, but is not applicable in practical engineering. In this paper, we further propose a random-sampling least-squares flow-estimation algorithm based on the median deviation. This method, called as RMLS, can obtain the optimal solution by minimizing the median value of the deviation, avoiding the setting of the error threshold in RLS, and distinguishing the inner and outer points through adaptive iteration. In this paper, Equations (17) and (18) are used to intelligently distinguish the inlier and outlier points [22]:

(17) $$\hat{\sigma }=1.4826\left(1+\frac{5}{a-n}\right)\min\left(med\left(D_{i}\right)\right)$$

where a denotes the number of the total data-points set, n is the number of data points at the least for LS estimation (in this study, n=4), $D_{i}$ is the distance calculated according to Equation (13), $med\left(D_{i}\right)$ is the median of the total points, and $\min\left(med\left(D_{i}\right)\right)$ is the minimum value of $med\left(D_{i}\right)$ in iterations. Thus, we can obtain dynamic $\hat{\sigma }$ in continuous iteration; using $\hat{\sigma }$, we can distinguish the inlier and outlier points as follows:

(18) $$N_{i}=\{\begin{array}{c} 1\;\;\;\;\;\;\;\frac{\left|\begin{array}{c} D_{i} \end{array}\right|}{\hat{\sigma }}\leq 2.5\\ 0\;\;\;\;\;\;\;\frac{\left|\begin{array}{c} D_{i} \end{array}\right|}{\hat{\sigma }}>2.5 \end{array}$$

where N=1 represents the inner point. The RMLS method divides the inner and outer points by using the distance median value instead of the a priori distance threshold value, thus enhancing the adaptability of the algorithm and avoiding the disadvantage that the RLS method needs to constantly adjust the threshold value.

The specific sketch of RMLS-based flow estimation algorithm is as Algorithm 3.
**Algorithm 3: RMLS-based flow estimation****Input:** Measured velocity matrix, V, and profiled average velocity vector, $V_{M}$. **Calculation process:** 1. For the t-th iteration, randomly generate the subset data, namely $V\left(s_{t}\right)$ and $V_{M}\left(s_{t}\right)$; 2. According to Equation (7), calculate the $\hat{W}\left(s_{t}\right)$; 3. According to Equation (13), calculate the distance, D, of the total data; 4. According to Equation (17), calculate the value of $\hat{\sigma }$; 5. According to Equation (18), statistically determine the number of inliers, namely $n\left(s_{t}\right)$; 6. According to Equation (16), calculate the maximum sampling iteration times, k; 7. If iteration times t<k, then count the maximum $n\left(s_{i}\right)$ and corresponding subset data; 8. Repeat steps (1) to (7) at t=k to obtain the optimal subset data, $V\left(s_{i}\right)$ and $V_{M}\left(s_{i}\right)$; 9. Based on the selected optimal subset, according to Equation (7), calculate the $\hat{W}$. **Output:** Velocity weight coefficient vector resolution, $\hat{W}$.

## 4. Results and Discussion

In this study, a double-bend pipe was taken as the research object. The double-bend outlet is located at x = 10 cm, and the measurement surface is set at x = 15, 50, and 300 cm. The fluid velocity in most engineering pipelines is within 1–7 m/s; as such, CFD software was used to collect 25 sets of data (each set includes four channel velocities and one plane average velocity) that were used for four optimization algorithms. We simulated four Gaussian noises, mainly considering four ultrasonic channels; however, the number of the Gaussian noise is not definite; it is neither too small nor too large. In the specified simulated scheme in the study, we randomly generated four Gaussian noises for 25 speeds, i.e., $v_{M}$ in Equation (1), i=randperm(25,4), where i=randperm(n,k) returns a row vector containing k unique integers selected randomly from 1 to n. For each speed needing to add Gaussian noise, any one of the four channels, namely $v_{ij}$, j=randperm(4,1), can satisfy ${\hat{v}}_{ij}=v_{ij}+GN\left(u,sigma\right)$. In the study, u=2, and sigma=1.

In this work, the relative error (RE) is taken as the main accuracy measurement standard and is calculated by using Equation (19):

(19) $$\mathrm{RE}=\frac{v_{M}-v_{r}}{v_{r}}\times 100\%$$

where $v_{r}$ is the true value of the average section velocity derived from the CFD software, and $v_{M}$ is the value of average section velocity calculated according to Equation (1). The relative error can accurately indicate the approximation degree between the average value of section velocity calculated by the flow algorithm and the true value.

**Figure 7:**
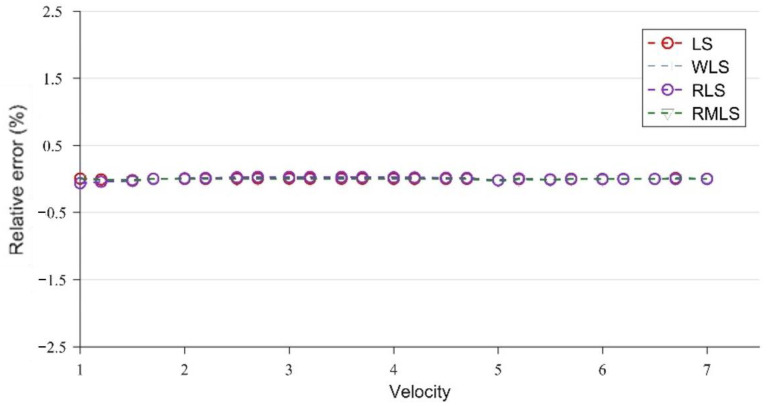
Results of LS, WLS, RLS, and RMLS without noise at x = 15 cm.

**Figure 8:**
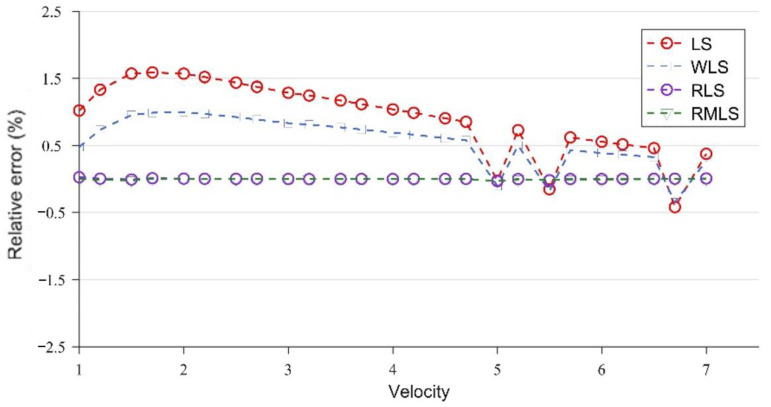
Results of LS, WLS, RLS, and RMLS with noise at x = 15 cm.

Figure 7 and Figure 8 are broken-line diagrams of the flow measurement of LS, WLS, RLS, and RMLS at x = 15 cm, under the conditions without and with noise, with the relative error as the accuracy measurement standard.

At x = 15 cm, the velocity profile has obvious structural characteristics, which are well captured by the four optimization algorithms, so the error of the parameters obtained under the four algorithms is very close to the true value. However, after adding Gaussian noise, the results of the four algorithms are significantly different. WLS is slightly better than LS, but both of them have large errors. By contrast, the two algorithms (RLS and RMLS) based on the random consistent sampling method have good accuracy and good noise resistance.

To confirm the validity of the results, we take the maximum relative error and average absolute percentage error of the LS, WLS, RLS, and RMLS algorithms at x = 15 cm for comparison (Table 1 and Table 2). The expression of the average absolute percentage error (MAPE) is as follows (18):

(20) $$\mathrm{MAPE}=\frac{1}{N}\overset{N}{\underset{i=1}{\sum }}\left|\begin{array}{c} \frac{v_{Mi}-v_{r}}{v_{r}} \end{array}\right|\times 100\%$$

As shown in Table 1 and Table 2, the four algorithms can achieve good accuracy in the absence of noise. The average absolute error is kept at the same level, and the maximum and minimum relative errors are not much different. With noise, the two methods based on random sampling (RLS and RLMS) have significant advantages and can still maintain the same accuracy level as that without noise. However, LS and WLS do not have such stability. The maximum and minimum relative errors and the average absolute percentage errors are greatly increased, and the accuracy is sharply reduced.

**Figure 9:**
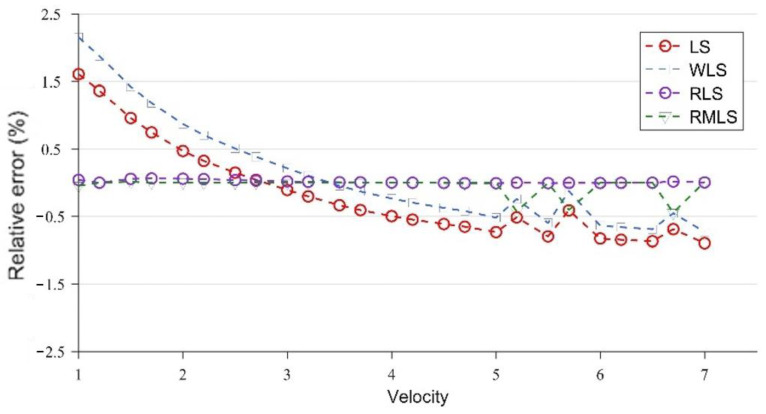
Results of LS, WLS, RLS, and RMLS with noise at x = 50 cm.

**Figure 10:**
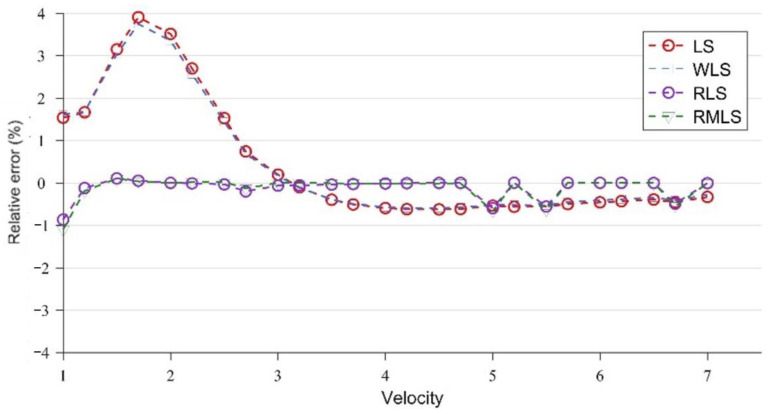
Results of LS, WLS, RLS, and RMLS with noise at x = 300 cm.

**Figure 11:**
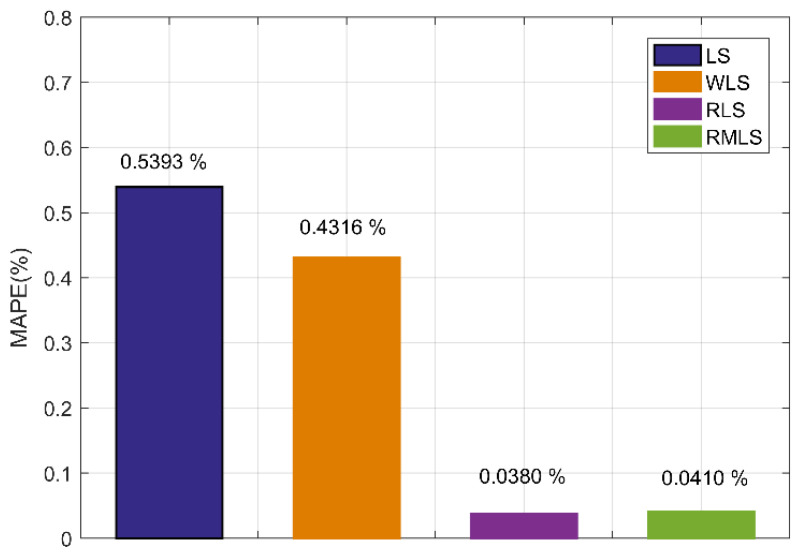
MAPE of four algorithms of 10,000 Monte Carlo simulation experiments at x = 50 cm.

At x = 15 cm, the flow field is severely deformed after the double bend. If the algorithm used is applicable to a symmetrical and fully developed flow, then it will inevitably cause huge errors. However, from another perspective, the velocity profile after the double bend has a very strong structure. The parameters calculated by the optimization algorithm can be adapted to flow measurement in almost all engineering speed ranges at the fixed position. Figure 7 and Table 2 confirm this finding. After coming out from the double bend, the velocity-profile deduction will have different changes at different speeds. From the results of the CFD calculation, this change is not obvious, and the profile structure and shape are similar at the fixed position. In this paper, the velocity profiles at the positions of x = 15, 50, and 300 cm are studied. As shown in Figure 4, Figure 5 and Figure 6, the structure and similarity of the velocity profiles at x = 15 and 50 cm are stronger but slightly weaker at x = 300 cm. However, the velocity distribution is relatively uniform at this time, and the high-accuracy results can still be obtained. At x = 50 and 300 cm, a group of noise is randomly added. The relative errors measured by the four algorithms are shown in Figure 9 and Figure 10:

As shown in Figure 9 and Figure 10, although the accuracy of RLS and RMLS at x = 50 and 300 cm is lower than that at x = 15 cm, they still have considerable accuracy and noise resistance. The evolution of the velocity profile is not only related to the location, but also to the inlet velocity. The location factor plays a decisive role near the elbow, for which the velocity profiles of different velocities have a strong structure. However, the role of the velocity factor gradually appears far from the elbow, weakening the similarity of the profiles of different velocities. At these locations, the symmetry is good, and the flow fields are uniform, so the algorithm still has a certain accuracy, but it is not as good as the near one. LS and WLS are sensitive to noise, resulting in a sharp drop in accuracy, and WLS cannot effectively reduce the measurement error at this time. However, RLS and RMLS still have considerable accuracy and noise resistance. Considering the random uncertainty of noise, we further verified the correctness of the experiment. We took x = 50 cm as an example to conduct 10,000 Monte Carlo simulation experiments and determined the average absolute percentage error of the four methods. The experimental results are shown in Figure 11.

In Figure 11, we can see that RLS and RMLS have significant advantages in the presence of noise. They have good accuracy, but RLS is very sensitive to the setting of the threshold, which needs repeated experiments to obtain the optimal value. By contrast, RMLS is adapted to distinguish the inner and outer points by minimizing the median deviation, so it does not need to manually set the error threshold, thereby improving the adaptability of the algorithm.

## 5. Conclusions

In this study, the flow field of a double-bend pipe, which is common in engineering, was taken as the research object. The detection surfaces were set at x = 15, 50, and 300 cm. Two optimization algorithms (RLS and RMLS) based on the random-consistent-sampling method were proposed. The results confirm that RLS and RMLS have higher accuracy and better noise resistance than the traditional LS and WLS. The traditional flow algorithm for the multichannel ultrasonic flowmeter is mostly used for ideal flow field with symmetric velocity profile. The accuracy of the flow field with severe deformation will drop sharply. In this work, the measurement error of x = 15 cm is very small, even lower than the flow measurement error at x = 300 cm. The most important reason is that the flow field at the pipe just out of the double bend has a strong structure, and the shape of the velocity profile is highly similar at any velocity. Therefore, a flow algorithm can be carried out for the pipe immediately out of the double bend to obtain high-precision measurement results. From the perspective, the flow field can be adjusted to the flow field with a specific structure by using specific equipment instead of returning to the ideal state, using a rectifier, and the corresponding flow algorithm is then applied to improve the measurement accuracy.

In this paper, three sections were studied (x = 15, 50, and 300 cm). From Figure 4, Figure 5 and Figure 6, we can see that the velocity profiles of different velocities at the same location are similar to each other, and this is the basis of the significance of this research. However, the deduction of the flow field is related to time and space. With the increasing velocity and distance, this similarity will have to be reduced. To further expand the research scope and the accuracy of the flow algorithm, we cannot only take the position as a variable but also introduce the velocity variable in combination with fluid mechanics to further improve the accuracy of the algorithm.

## Figures and Tables

**Table 1:** Accuracy statistical indices of four algorithms at x = 15 cm (without noise).

	LS	WLS	RLS	RMLS
Maximum relative error (%)	0.020	0.020	0.028	0.032
Mean absolute percentage error (%)	0.0052	0.0052	0.0049	0.0048

**Table 2:** Accuracy statistical indices of four algorithms at x = 15 cm (with noise).

	LS	WLS	RLS	RMLS
Maximum relative error (%)	1.53	1.03	0.027	0.067
Mean absolute percentage error (%)	0.95	0.62	0.0057	0.0049
